# *Sargassum horneri* as a Functional Food Ameliorated IgE/BSA-Induced Mast Cell Activation and Passive Cutaneous Anaphylaxis in Mice

**DOI:** 10.3390/md18120594

**Published:** 2020-11-26

**Authors:** Eui Jeong Han, Hyun-Soo Kim, Kalu Kapuge Asanka Sanjeewa, Kyungsook Jung, Youngheun Jee, You-Jin Jeon, Ilekuttige Priyan Shanura Fernando, Ginnae Ahn

**Affiliations:** 1 Research Center for Healthcare and Biomedical Engineering, Chonnam National University, Yeosu 59626, Korea; iosu5772@jnu.ac.kr; 2 Department of Food Technology and Nutrition, Chonnam National University, Yeosu 59626, Korea; 3 National Marine Biodiversity Institute of Korea, Janghang-eup, Seocheon 33662, Korea; gustn783@mabik.re.kr; 4 Department of Marine Life Science, School of Marine Biomedical Sciences, Jeju National University, Jeju 63243, Korea; asanka.sanjeewa001@jejunu.ac.kr (K.K.A.S.); youjinj@jejunu.ac.kr (Y.-J.J.); 5 Biomaterials Research Center, Korea Research Institute of Bioscience and Biotechnology, Jeonbuk 56212, Korea; jungks@kribb.re.kr; 6 Department of Veterinary Medicine and Veterinary Medical Research Institute, Jeju National University, Jeju 63243, Korea; yhjee@jejunu.ac.kr; 7 Interdisciplinary Graduate Program in Advanced Convergence Technology & Science, Jeju National University, Jeju 63243, Korea; 8 Department of Marine Bio-Food Sciences, Chonnam National University, Yeosu 59626, Korea; shanurabru@jnu.ac.kr; 9 Control Center for Aquatic Animal Diseases, Chonnam National University, Yeosu 59626, Korea

**Keywords:** *Sargassum horneri*, bone marrow-derived cultured-mast cells (BMCMCs), immunoglobulin e, passive cutaneous anaphylaxis (PCA), anti-allergic effect

## Abstract

*Sargassum horneri* (*S. horneri*), an edible brown alga, has been proposed as a functional food with an improvement effect on abnormal skin immune responses. The present study investigates the anti-allergic effect of an ethanol extract from *S. horneri* (SHE) on immunoglobulin E (IgE)/bovine serum albumin (BSA)-mediated activation in bone marrow-derived cultured-mast cells (BMCMCs) and passive cutaneous anaphylaxis (PCA) reaction in mice. SHE markedly and dose-dependently suppressed the degranulation of BMCMCs by reducing the β-hexosaminidase and histamine release without cytotoxicity. In addition, SHE significantly decreased the FcεRI expression on the surface of BMCMCs and its IgE binding. Moreover, SHE reduced the mRNA expression and the production of allergic cytokines; interleukin (IL)-1β, IL-4, IL-5, IL-6, IL-10, IL-13; interferon (IFN)-γ and/or tumor necrosis factor (TNF)-α; and a chemokine, thymus and activation-regulated chemokine (TARC), by suppressing the activation of Src-family kinases and nuclear factor (NF)-κB signaling. In further study, the application of SHE reduced the PCA reaction in an IgE/BSA-induced type I allergic mice model. Taken together, we suggest that SHE has an anti-allergic effect in type I allergic responses.

## 1. Introduction

Natural functional food has acquired growing attention, as its consumption offers long-lasting health benefits [1]. Instead of relying on synthetic drugs, there is a growing social tendency for enhancing physiological well-being against diseases such as dysregulated immune responses, hypertension, and obesity via increasing the consumption of natural foods [2]. In recent years, the rise in the prevalence of allergies has been particularly noticeable in developed Western countries. Though hereditary predisposition is a major cause of increased susceptibility, genetics alone cannot clarify the increased prevalence of allergies. The rise of allergies is hypothesized to be due to increased hygiene, where the immune system fails to identify harmless and harmful irritants [3]. Among numerous allergy responses, the majority are categorized under type 1 allergic reactions, which account for nearly 90% of all reported cases [4]. The onset of type 1 allergic reaction follows a chain of chemical reactions and biological responses against certain harmless allergens [5]. These allergies lead to various diseases, including allergic rhinitis, atopic dermatitis, allergic asthma, and eczema, which are the commonly found immunoglobulin E (IgE)-mediated allergic responses [5,6,7].

Mast cells and basophils play a crucial role in the pathogenesis of type 1 allergy reactions. Activated mast cells release various allergic mediators such as lipid mediators (leukotrienes and prostaglandins), granular contents (β-hexosaminidase and histamine), and allergic cytokines (interleukin (IL-4), IL-5, IL-6, IL-13, interferon (IFN)-γ, and tumor necrosis factor (TNF)-α). The high-affinity immunoglobulin E receptor (FcεRI) existing on the surface of mast cells binds with IgE and leads to the degranulation and allergic cytokine production of mast cells [8]. At these points, inhibiting mast cell activation via a reduction in FcεRI expression and its IgE binding is a crucial target for the improvement of type I allergic diseases. Moreover, many drugs have been used for the remedy of type I allergic diseases for the last few decades, but they are associated with numerous side-effects, such as irritation, stomach upset, drowsiness, etc. [9]. Therefore, many researchers have focused on the use of naturally available bioactive compounds in the regulation of allergies to minimalize harmful side-effects occurring from over-the-counter drugs, which are highly addictive.

Natural products of marine algae report countering various diseases such as inflammation, oxidative stress, and diabetes [10]. *Sargassum horneri* (*S. horneri*), an abundant seaweed, is a food delicacy in China, Japan, and Korea as a medicinal food rich in nutritional value for preparing soups and side dishes [11]. Recently, the Ministry of Food and Drug Safety of Korea (MFDS) has paid special attention to utilizing *S. horneri*, while exploring its health beneficial functionalities to promote developing industrial processes to manufacture essential consumables [12]. Based on numerous investigations, its extracts and derives such as (−)-loliolide, chromene, phytol, fucosterol, saringosterol, β-sitosterol, and polysaccharides have reported possessing various bioactivities encompassing protective effects against lung damage, inflammation, atopy, and oxidative stress [13]. Although there are numerous studies related to the bioactivities of *S. horneri*, few studies have explored its effects on mediating mast cell activation-mediated allergic responses.

The present study was undertaken to investigate if *S. horneri* ethanol extract (SHE) suppresses IgE-mediated allergic responses in mouse bone marrow-derived cultured mast cells (BMCMCs) and a passive cutaneous anaphylaxis (PCA) mice ear model.

## 2. Results

### 2.1. SHE Inhibited the Degranulation of IgE/BSA-Activated BMCMCs without Cytotoxicity

**Figure 1:**
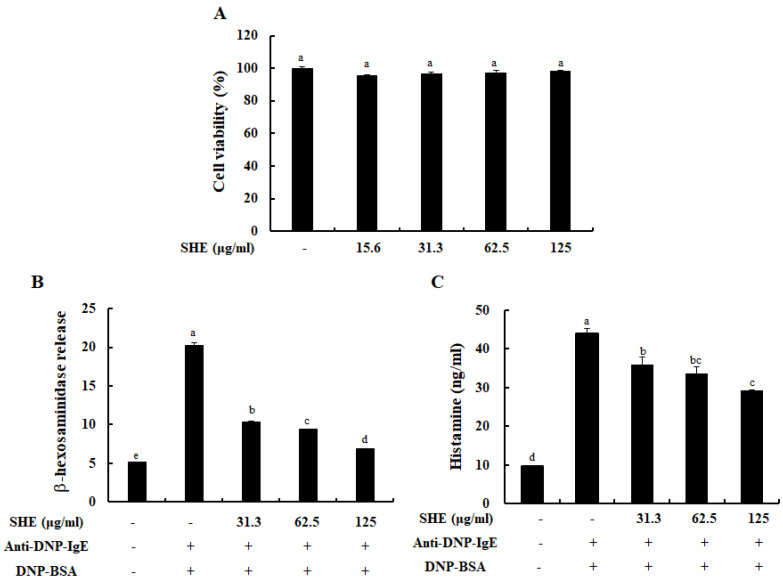
Cytotocompatibility of SHE and its effect on BMCMCs degranulation. (**A**) Effects of SHE on BMCMCs viability. Effects of SHE on (**B**) β-hexosaminidase and (**C**) histamine release in immunoglobulin E (IgE)/bovine serum albumin (BSA)-stimulated BMCMCs. The reproducibility of the results was confirmed by triplicate determinations (*n* = 3). a–d: bars with different letters represent significant differences (*p* < 0.05).

To evaluate whether SHE has cytotoxicity on BMCMCs, we performed an MTT assay. As Figure 1A indicates, SHE did not show any cytotoxicity at the used all concentrations. Next, we examined the effect of SHE on the degranulation of BMCMCs, known as a biomarker of the activation of mast cells. Figure 1B,C exhibited that the stimulation of IgE/BSA markedly increased the release of β-hexosaminidase and histamine from BMCMCs, whereas it was dose-dependently reduced by the pre-treatment of SHE. With these results, we identified that SHE inhibited the degranulation of BMCMCs activated by IgE/BSA without cytotoxicity.

### 2.2. SHE Reduced the Expression of FcεRI on the Surface of BMCMCs and Its IgE Binding

**Figure 2:**
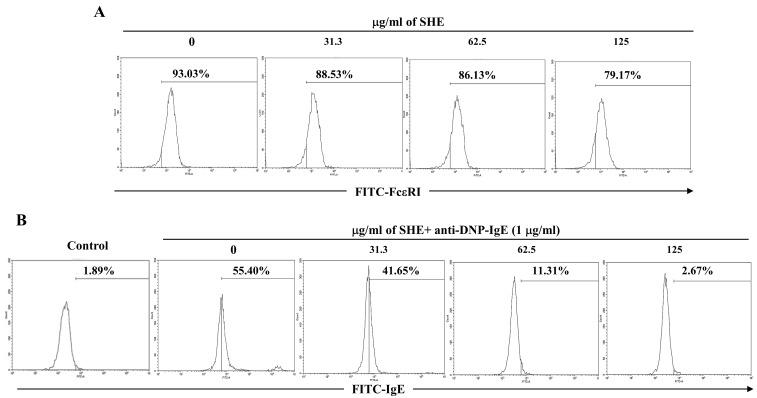
Effects of SHE on FcεRI expression and its IgE binding on the surface of BMCMCs. (**A**) Surface expression levels of FcεRI and (**B**) expression levels of IgE bound to the FcεRI of BMCMCs. The reproducibility of the results was confirmed by triplicate determinations (*n* = 3).

To check whether SHE affects the expression of FcεRI on the surface of BMCMCs and its IgE binding, we performed a flow cytometry analysis. As shown in Figure 2A, SHE treatment dose-dependently decreased the FcεRI expression on the surface of BMCMCs. In addition, the stimulation of IgE markedly increased the expression of IgE bound to FcεRI on the surface of BMCMCs compared to the non-stimulated cells (Figure 2B). In contrast, the treatment of SHE effectively and dose-dependently decreased the binding of IgE on IgE-stimulated BMCMCs (Figure 2B). In particular, 125 µg/mL of SHE considerably reduced the expression of IgE bound to FcεRI on the surface of BMCMCs, compared to only IgE-stimulated cells. This result indicates that the role of SHE on the FcεRI expression and its IgE binding led to the inhibition of BMCMCs degranulation.

### 2.3. SHE Down-Regulated the mRNA Expression Levels of Allergic Cytokines and a Chemokine in IgE/BSA-Stimulated BMCMCs

**Figure 3:**
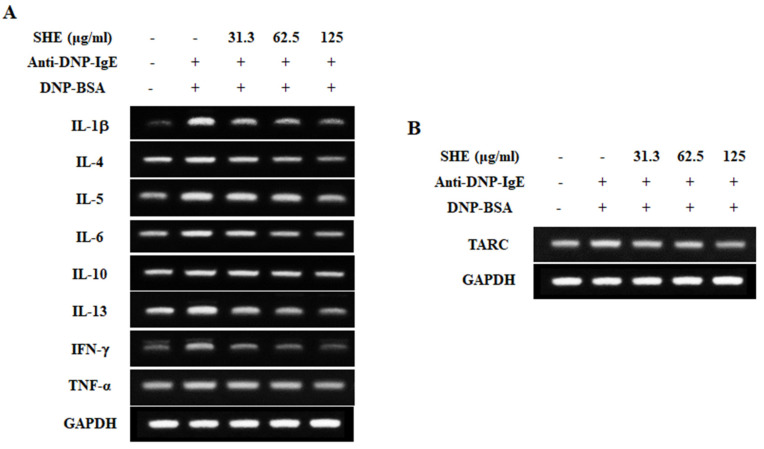
Role of SHE in the mRNA expression levels of allergic cytokines (**A**) and a chemokine (**B**) in IgE/BSA-stimulated BMCMCs. The reproducibility of the results was confirmed by triplicate determinations (*n* = 3).

RT-PCR analysis was performed to investigate the effect of SHE on the mRNA expression levels of pro-inflammatory cytokines and a chemokine in IgE/BSA-stimulated BMCMCs. Figure 3A exhibited that IgE/BSA stimulation markedly increased the mRNA expression levels of allergic cytokines such as IL-1β, IL-4, IL-5, IL-6, IL-10, IL-13, IFN-γ, and TNF-α, compared to the non-stimulated cells. Interestingly, they were effectively down-regulated by the pretreatment of SHE. In addition, SHE led to a reduction in the mRNA expression level of TARC increased by the IgE/BSA stimulation in BMCMCs (Figure 3B). From these results, we indicate that SHE has an anti-allergic effect by modulating the mRNA expression levels of allergic cytokines and a chemokine in IgE/BSA-stimulated BMCMCs.

### 2.4. SHE Reduced the Production of Allergic Cytokines in IgE/BSA-Stimulated BMCMCs

**Figure 4:**
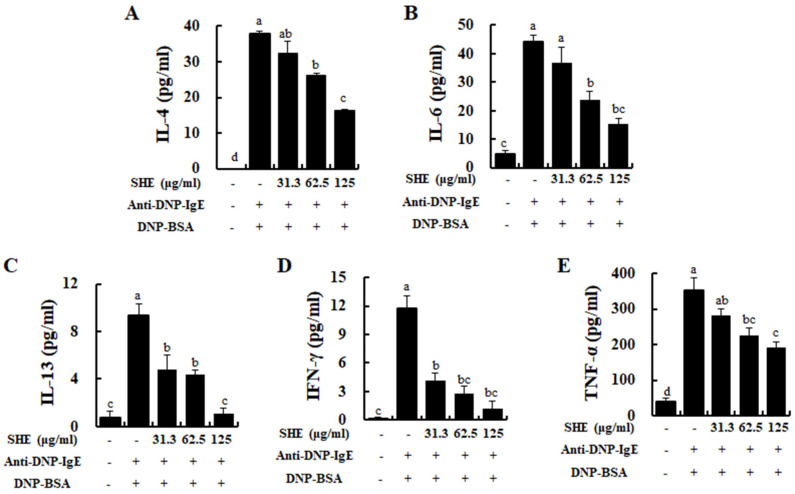
Effects of SHE on the production of allergic cytokines in IgE/BSA-stimulated BMCMCs. (**A**) IL-4, (**B**) IL-6, (**C**) IL-13, (**D**) IFN-γ, and (**E**) TNF-α. Cytokine levels were measured by commercial ELISA kits. The reproducibility of the results was confirmed by triplicate determinations (*n* = 3). a–d: bars with different letters represent significant differences (*p* < 0.05).

To evaluate the effect of SHE on the production of allergic cytokines in IgE/BSA-stimulated BMCMCs, we performed ELISA. As shown in Figure 4, IgE/BSA stimulation significantly increased the productions of allergic cytokines such as IL-4, IL-6, IL-13, IFN-γ, and TNF-α in BMCMCs. In contrast, they were significantly and dose-dependently reduced by the treatment of SHE. This result demonstrates that SHE has an anti-allergic effect by reducing the production of allergic cytokines in IgE/BSA-stimulated BMCMCs as well as decreasing their mRNA expression.

### 2.5. SHE Inhibited the Protein Expression of Src-Family Kinases and NF-κB Signaling Pathway in IgE/BSA-Stimulated BMCMCs

**Figure 5:**
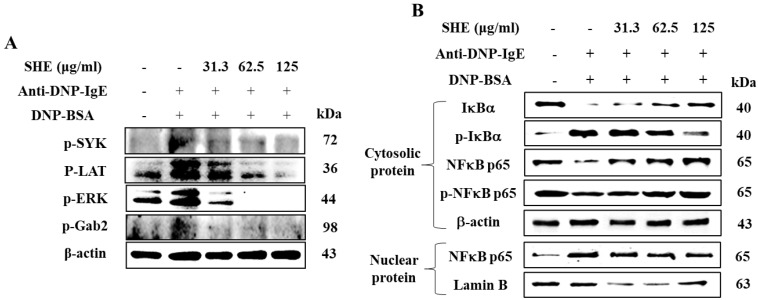
Roles of SHE on the Src-family kinases (**A**) and the NF-κB signaling pathway (**B**) in IgE/BSA-stimulated BMCMCs. The reproducibility of the results was confirmed by triplicate determinations (*n* = 3).

We examined the effect of SHE on the signaling of Src-family kinases and NF-κB in IgE/BSA-stimulated BMCMCs by Western blot analysis. As shown in Figure 5A, the IgE/BSA stimulation increased the expression levels of phosphorylated SYK, LAT, Gab2, and ERK in the Src-family kinases in BMCMCs, whereas they were markedly decreased by the SHE pretreatment. In further evaluation, the IgE/BSA stimulation induced the degranulation and phosphorylation of IκBα and NFκB p65 in the cytosol as well as the translocation of NFκB-p65 into the nucleus (Figure 5B). Interestingly, SHE pre-treatment effectively modulated in a dose-dependent manner. With these results, we suggest that SHE effectively modulated the signaling of Src-family kinases and NF-κB in IgE/BSA-stimulated BMCMCs.

### 2.6. SHE Decreased PCA Reaction in IgE/BSA-Stimulated Mice

**Figure 6:**
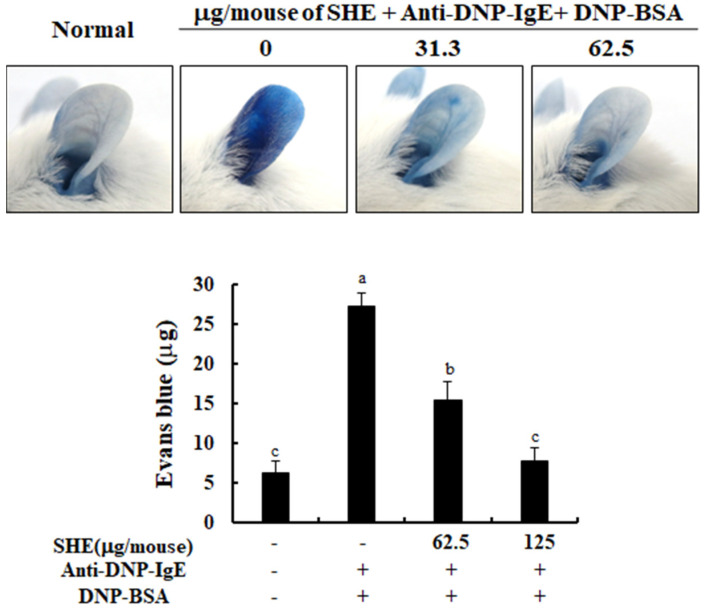
Effect of SHE on IgE/BSA-mediated passive cutaneous anaphylactic (PCA) reaction in BALB/c mice. The amounts of Evans blue dye extravagated from mice ear skin are analyzed. The reproducibility of the results was confirmed by triplicate determinations (*n* = 5). a–c: bars with different letters represent significant differences (*p* < 0.05).

A PCA mice ear model was used to investigate the effects of SHE on mast cell-dependent allergic responses in an in vivo model. As shown in Figure 6, the stimulation with IgE/BSA caused the development of a strong PCA concomitant with the increased Evans blue dye amount, compared to the non-stimulated mice. Interestingly, the application of SHE led to a reduction in the Evans blue amount extracted from mice ear skin in an IgE/BSA stimulation-induced PCA mice ear model. With this result, we indicate that the anti-allergic effect of SHE on the activation of mast cells affected the improvement of the PCA reaction in in vivo models.

## 3. Discussion

The present study investigated the anti-allergic effect of the ethanol extract of *S. horneri* (SHE) on the IgE-mediated allergic responses in BMCMCs and a PCA mice ear model. We revealed that SHE ameliorated the IgE/BSA-stimulated mast cell activation by reducing the release of β-hexosaminidase and histamine and the expressions and production of allergic mediators via the regulation of FcεRI expression and its IgE binding, as well as the inhibition of Src-family kinases and NF-κB signaling

Mast cells are a part of innate and adaptive immune responses and contribute to various pathophysiological conditions, such as allergic or immediate hypersensitivity responses [14]. In addition, mast cells play a crucial role in the progression of allergy reactions via the degranulation process predominantly caused by the stimulation of IgE/BSA. The degree of degranulation reflects the levels of mast cell activation [15]. Normally, the release of β-hexosaminidase and histamine has been reported as a key biomarker for the degranulation of the activated mast cells [16,17,18]. Interestingly, our result exhibited that SHE inhibited the degranulation of BMCMCs by reducing the release of β-hexosaminidase and histamine induced by the IgE/BSA stimulation.

Normally, FcεRI, a surface receptor has three different subunits (α chain, β chain, and γ chain) in mast cells, and among them its α chain subunit directly binds to IgE, followed by the activation of mast cells [19,20]. After the attachment of a specific allergen to each of the adjacent IgE molecules, by forming the cross-linkage, it makes the antigen-antibody complex, which provides the signal for mast cell activation, especially degranulation [19,21]. FcεRI-IgE complexation causes the activation of several downstream pathways that initiate allergic and inflammatory process by eliciting mast cell degranulation with a rapid release of preformed vasoactive amines such as histamine and serotonin [22]. Many researchers have evaluated the inhibitory effects of natural components on the FcεRI expression levels and its IgE binding in mast cells as well as their degranulation [23,24]. Our results also reported that SHE treatment dose-dependently decreased the FcεRI expression on the surface of BMCMCs. Additionally, a flow cytometry assay and molecular docking analysis exhibited that the major compound of SHE, loliolide, decreased the FcεRI expression on the surface of BMCMCs by binding to the α chain subunit of FcεRI (data not shown). These results suggest that loliolide might contribute to the SHE’s ability in the down-regulation of the FcεRI expression as it binds to the α chain subunit of FcεRI in BMCMCs. Additionally, SHE induced a reduction in the FcεRI expression, and its IgE binding finally resulted in anti-allergic effects. Indeed, the inhibitory capacities of SHE in the FcεRI expression and its IgE binding effectively contributed to the reduction in the degranulation of the activated BMCMCs. Moreover, in the previous study, the researcher has reported that β-hexosaminidase and histamine releases can affect the production and expression of allergic cytokines, including IL-1α, IL-2, IL-3, IL-4, IL-5, IL-6, IL-13, GM-CSF, TGF-β, TNF-α, and IFN-γ and a chemokine including TARC through the subsequent induction of numerous molecular mediators in IgE/BSA-activated BMCMCs [16]. Generally, IL-1β promotes hematopoiesis and the release of IL-10, while IL-4 targets Th2 cells to induce differentiation [25]. IL-4 and IL-13 play an important role in mediating allergic reactions which can promote the induction of IgE syntheses and the development of mast cells [17,26]. Particularly, IL-4 is the initial cytokine secreted from mast cells after IgE/BSA stimulation and activates secondary cytokines such as IL-5, IL-6, and IL-13 [26,27]. IL-6 had been defined to modulate the adaptive immune response during early T cell activation, and TNF-α, which is a major effector cytokine in allergic reaction, improves mediator expression and cytokines in mast cells [28,29]. Especially, TNF-α is produced during the degranulation process of a mast cell, and it leads to chronic inflammation by promoting eosinophil survival while mobilizing both neutrophils and T cells [30]. Interferon γ (IFN-γ) promotes Th1 cell differentiation and epithelial apoptosis in the mucosa and skin [25]. TRAC is another important mediator that exacerbates allergy reactions with the infiltration of eosinophils and Th2 cells, as well contributing to various diseases induced by mast cell activation [31]. Based on the above points, the inhibition of these allergic cytokines and a chemokine is one of the major indicators for the improvement of allergic responses. In our results, we identified that the IgE/BSA stimulation significantly increased the mRNA expression and production of allergic cytokines such as IL-1β, IL-4, IL-5, IL-6, IL-10, IL-13, IFN-γ, and TNF-α, whereas they were dose-dependently attenuated by SHE. Additionally, SHE down-regulated the mRNA expression level of a chemokine, TARC, in IgE/BSA-activated BMCMCs. With these results, this study indicates that SHE suppressed the allergic reaction by reducing the expression and production of allergic cytokines and a chemokine as well as the degranulation by modulating the expression of FcεRI and its IgE binding in IgE/BSA-activated BMCMCs.

The signal for mast cell degranulation proceeds via a complex web of signaling pathways that involve many secondary signaling molecules [32]. The signaling is initiated through the phosphorylation of immunoreceptor tyrosine-based activation motifs in the tails of the FcεRI and subunits by Src-family protein tyrosine kinases [22]. These phosphorylated immunoreceptors act as binding sites for secondary signaling molecules such as LAT and Gab2 [33]. ERK is a secondary signaling molecule that belongs to the NF-κB pathway and is related to the activation of mast cells [24]. As evidenced by the present results, the expression levels of the signaling of Src-family kinases, including p-Syk, p-LAT, p-Gab2, and p-ERK, were significantly increased upon IgE/BSA stimulation, whereas they were decreased by SHE treatment.

Generally, the NF-κB family consists of NF-κB p50, NF-κB p52, NF-κB p65, relB, and c-rel mediators. Cytosolic NF-κB transcription factors are heterodimers in the Rel homology family (including p50 and p65) [34]. Under physiological conditions, IκB, which prevents their nuclear translocation, is degraded and phosphorylated [19]. The phosphorylation of IκB breaks down the complexes IκB-p50 and IκB-p65, which enables the translocation of Rel proteins to the nucleus. Eventually, the Rel proteins bind with target genes at κB-binding sites, inducing the transcription of inflammatory mediators (iNOS, PGE2, and COX-2) and pro-inflammatory cytokines (TNF-α, IL-1β, and IL-6) [34]. Previous reports have explained that Src-family kinase signaling activates the transcription factor, NF-κB, and promotes the release of allergic cytokines [22]. The above molecular mediators are responsible for activating the NF-κB signaling pathway, which promotes allergic or inflammatory cytokines release. According to the present evaluations, SHE effectively reduced the phosphorylation of IκBα and NF-κB p65 as well as its translocation into the nucleus elevated by IgE/BSA-stimulation. These observations suggest that SHE led to a reduction in allergic mediators inhibiting NF-κB signaling by regulating the activation of Src-family kinases in IgE/BSA-stimulated BMCMCs. Moreover, this study suggests that SHE inhibited the mast cell activation induced by IgE/BSA stimulation by down-regulating the binding of IgE and FcεRI as well as the Src-family kinases and NF-κB signaling and has an anti-allergic effect in BMCMCs.

The PCA mice model is a well-established method to investigate the effects of drugs or natural materials on mast cell-dependent allergic responses mediated by the binding of IgE and FcεRI [35]. Our results showed that the stimulation of IgE/BSA containing Evans blue caused a marked PCA reaction in mice ear skin, compared to the non-stimulated mice model, whereas it was dose-dependently reduced by the application of SHE (Figure 6). These data demonstrated has the anti-allergic effect of SHE against PCA reaction induced by IgE/BSA stimulation in an in vivo model. With the above points, this study suggests that the role of SHE in attenuating mast cell-dependent allergic responses, particularly the binding of IgE with FcεRI, led to its anti-allergic effect against PCA reaction in mice.

## 4. Materials and Methods

### 4.1. Chemicals and Sample

Alpha Minimum Essential Medium Eagle (α-MEM), penicillin-streptomycin, and fetal bovine serum (FBS) were purchased from Gibco BRL (Burlington, ON, Canada). Dimethyl sulfoxide and 3-(4-5-dimethyl-2yl)-2-5-diphynyltetrasolium bromide (MTT) were obtained from Sigma-Aldrich (St, Louis, Mo, USA). Trizol reagent was purchased from the molecular research center (Montgomery, OH, USA). cDNA synthesis kit was purchased from Promega Co. (Madison, WI, USA). Other chemicals and regents used were of the highest grade available commercially. The SHE used in this study was equal to the sample prepared from *S. horneri* in the previous study [11,12].

### 4.2. Mice

The C57/BL6 and BALB/c mice (8 weeks) were purchased from Orientbio, Inc. (Seongnam, Korea) and housed under a controlled temperature (22 ± 2 °C) and humidity (22 ± 10%), with light at 12 h light/12 h dark. The institutional ethical committee approved these experiments (Approval No. CNU IACUC-YS-2019-4) and they complied with the “Principles of Laboratory Animal Care” guidelines (NIH publication No. 80-23, revised 1996).

### 4.3. Isolation of Bone Marrow-Derived Cultured-Mast Cells (BMCMCs) Form Mice

BMCMCs were obtained from the bone marrow of C57/BL6 mice according to the method previously described by this research [36]. The cells were cultured with α-MEM media (10% FBS, 1% penicillin-streptomycin) with 10% pokeweed mitogen-stimulated spleen cell-conditioned medium (PWM-SCM) and 0.2% mercaptoethanol (2-ME, Sigma-Aldrich, St, Louis, Mo, USA). The cells were cultured once a week for 8 weeks, and above 98% of the mast cells recognized as Giemsa staining positive were used for the experiments.

### 4.4. Measurement of Cytotoxicity in BMCMCs

The cytotoxicity of SHE was measured by a 3-(4-5-dimethyl-2yl)-2-5-diphynyltetrasolium bromide (MTT) assay. The cells (2 × 10^4^/well) were treated with the various concentrations of SHE (15.6–62.5 μg/mL) on BMCMCs. After 24 h, the cells were reacted with 15 μL of MTT stock solution (5 mg/mL) for 4 h. The formazan crystals were dissolved in 150 μL of dimethylformamide (DMSO) and measured at 540 nm using a microplate reader (Sunrise, Tecan Co. Ltd., Grödig, Austria).

### 4.5. Measurement of β-Hexosaminidase and Histamine Release

The effect of SHE on the β-hexosaminidase release from mast cells was evaluated according to the method reported by this research [37]. Briefly, BMCMCs (2 × 10^5^/well) pre-incubated with various concentrations of SHE for 2 h were sensitized by anti-DNP-IgE for 1 h and stimulated by DNP-BSA (LSL Japan Inc., Tokyo, Japan, 100 ng/mL). After 1 h, the supernatant was obtained from the cells and the cells were lysed with Tyrode’s buffer containing 0.5% Triton X-100. Then, 0.1 M sodium citrate substrate buffer (pH 4.5) containing 4-p-nitrophenyl-*N*-acetyl-β-d-glucosaminide 1.3 mg/mL was added to the supernatants and the cell lysates. The mixtures were reacted for 1 h at 37 °C. The reaction was terminated by adding 0.2 M glycine (pH 10.7). The absorbance of each mixture was measured at 405 nm using a microplate reader. Histamine release was evaluated using a Histamine EIA kit (Oxford Biomedical Research, Oxford, MI, USA).

### 4.6. Measurement of FcεRI Expression on the Surface of BMCMCs and Its IgE Binding

To confirm the effect of SHE on the surface FcεRI expression levels of BMCMCs, we performed flow cytometry analysis. BMCMCs (4 × 10^5^ cells/well) were treated with SHE for 6 h at 37 °C and blocked by anti-CD16/CD32 monoclonal antibody (Thermo Fisher Scientific, Rockford, IL, USA) for 20 min to prevent nonspecific binding of antibodies. Then, the cells were stained with fluorescein (FITC)-conjugated anti-FcεRI antibody (clone: MAR-1; eBioscience, San Diego, CA, USA).

Next, to examine the effect of SHE on the IgE binding on FcεRI of BMCMCs, we performed a flow cytometry analysis. BMCMCs (4 × 10^5^ cells/well) were treated with SHE for 2 h at 37 °C and sensitized by anti-DNP-IgE (1 μg/mL) for 4 h. The cells were blocked by anti-CD16/CD32 monoclonal antibody for 20 min and stained with FITC-conjugated anti-mouse IgE monoclonal antibody (clone: 23G3; eBioscience, San Diego, CA, USA). The fluorescently stained cells were analyzed by a CytoFLEX flow cytometer (Beckman coulter, Fullerton, CA, USA).

### 4.7. Measurement of the mRNA Expression of Allergic Cytokines and a Chemokine

To check the effect of SHE on the mRNA expression levels of allergic cytokines and a chemokine in the activated BMCMCs, a reverse transcription-polymerase chain reaction (RT-PCR) was carried out. BMCMCs (2 × 10^6^/well) incubated with SHE for 2 h were sensitized by anti-DNP-IgE (1 μg/mL) for 4 h and then stimulated by DNP-BSA (100 ng/mL) for 24 h. Total RNA isolated from BMCMCs by Trizol reagent was used for the cDNA synthesis using PrimeScript RT Reagent Kit (TaKaRa BIO INC, Shiga, Japan). PCR was carried out for 35 cycles using the respective primers indicated in our previous study [38]. PCR conditions were set for 5 min denaturing at 94 °C, 1 min annealing at 55–60 °C, and a 20 min extension phase at 72 °C in a TaKaRa PCR Thermal Cycler (TaKaRa Bio Inc., Otsu, Japan). The products of the PCR were electrophoresed on 1.5% ethidium bromide/agarose gels and visualized under UV transillumination (Vilber Lourmat, Marne la Uallee, France).

### 4.8. Measurement of Allergic Cytokines and a Chemokine Production

BMCMCs (2 × 10^6^/well) incubated with SHE for 2 h were sensitized by anti-DNP-IgE (1 μg/mL) for 4 h and then stimulated by DNP-BSA (100 ng/mL) for 24 h. The supernatants were used to analyze the production of allergic cytokines and a chemokine by mouse cytokine ELISA kits (Biolegend Inc., San Diego, CA, USA), following the manufacturer’s instructions.

### 4.9. Western Blot Analysis

BMCMCs (1 × 10^6^ cells/dish) were incubated with SHE for 2 h before anti-DNP-IgE (1 μg/mL) sensitization. After 4 h, the cells were treated with DNP-BSA (100 ng/mL) for 30 min and 15 min, respectively, for NF-κB proteins and Src-family kinases. Cytosolic and nucleic proteins were obtained from the cells with the NE-PER^®^ nuclear and cytoplasmic extraction kit (Thermo Scientific, Waltham, MA, USA). The protein concentrations of both cell lysates were measured using the BCA^TM^ protein assay kit according to the manufacturer’s instructions. Both protein samples were separated on 10% sodium dodecyl sulfate (SDS)-polyacrylamide gel electrophoresis (SDS–PAGE, Bio-Rad, Hercules, CA, USA) and transferred onto nitrocellulose membranes. The membranes were blocked in 5% non-fat dry milk in TBS-T (137 mM NaCl, 25 mM Tris–HCl, 2.65 mM KCl, 0.05% Tween 20, pH 7.4) and then incubated with the respective primary antibody, Syk, LAT, Gab2, p-ERK, IκBα, p-IκBα, p65, and p-p65 (1:1000 dilution, Cell Signaling Technology, Beverly, MA, USA) overnight at 4 °C. Subsequently, the membranes were incubated with the horseradish peroxidase (HRP)-conjugated secondary antibodies (anti-mouse IgG and anti-rabbit IgG, 1:5000, Cell Signaling Technology, Danvers, MA, USA) for 90 min. The bands were detected using an enhanced Super Signal West Femto Maximum Sensitivity Substrate (Thermo Scientific) reagents and analyzed using the NIH Image J software (Version No.1, US National Institutes of Health, Bethesda, MD, USA).

### 4.10. Measurement of Passive Cutaneous Anaphylaxis (PCA) in Mice

Next, we investigated the effect of SHE on anaphylactic reactions in an IgE/BSA-induced PCA mice model. Anti-DNP-IgE (500 ng) was intradermally injected into the ear surface of mice for 46 h and each ear was painted with SHE (62.5 and 125 μg/20 uL in saline). After 2 h, the mice were intravenously injected with DNP-BSA (10 mg) containing 4% Evans blue dye. The control group of mice was treated with saline instead of SHE. After 30 min, the ear tissues collected from the mice were soaked in 1 mL of formamide overnight at 64 °C. The amount of Evans blue dye extravagated from each ear tissue was calculated by the measurement of their absorbance at 620 nm.

### 4.11. Statistical Analysis

Data were analyzed using the SPSS package (Version 21, IBM, Armonk, NY, USA). Results are expressed as means ± standard error (SE). The mean values of the tail intensity from each treatment were compared using a one-way analysis of variance (ANOVA), followed by Duncan’s multiple range test. A *p*-value of less than 0.05 was considered significant.

## 5. Conclusions

Collectively, this study suggests that SHE elicits an anti-allergic effect via the improvement of the PCA reaction by suppressing mast cell activation, and it can be used for the development of a natural functional food supplement for ameliorating type 1 allergy reactions.
